# A Numerical Study on the Drug Release Process of Biodegradable Polymer Drug-Loaded Vascular Stents

**DOI:** 10.3390/polym17030420

**Published:** 2025-02-05

**Authors:** Shiyong Li, Yunbo Wei, Hongxia Li

**Affiliations:** 1 Dalian Rubber & Plastics Machinery Co., Ltd., Dalian 116036, China; lishiyong@dxs1907.cn (S.L.); weiyunbo@mail.dlut.edu.cn (Y.W.); 2 School of Mechanical Engineering, Dalian University of Technology, Dalian 116024, China

**Keywords:** biodegradable polymer, drug-loaded stents, drug release, finite element method

## Abstract

Biodegradable polymer drug-loaded vascular stents are a typical and promising application in the field of invasive interventional therapy. The drug release process of drug-loaded vascular stents, as well as the drug concentration in the vascular wall and its change process, will affect the therapeutic effect of vascular stents on vascular stenosis. As a drug carrier, the degradation properties of the polymer will affect the drug release process. In this study, the drug release process from the biodegradable polymer stent and the drug delivery process in vascular lumens and intravascular walls were studied by using 3D finite element method, with the effect of the biodegradation behavior of polymer on the drug release process being considered. The effects of the initial drug concentration, stent geometry, and polymer degradation rate on the drug release and delivery process were investigated. The results showed that the initial drug concentration and the thickness of the polymer stent significantly affected the drug concentration in the middle layer of the vessel wall, but the initial drug concentration had no effect on the drug release duration. The degradation of the polymer causes its porosity to change with time, which affects the drug diffusion in polymer, and further affects the drug concentration in the vessel wall. The three-dimensional structure of the stent can affect the blood flow in the blood vessel, resulting in drug deposition near the struts, especially near the intersection of the support struts and the bridge struts.

## 1. Introduction

Biodegradable polymers have a wide range of applications in biomedical fields. Biodegradable polymer drug-loaded vascular stents are a typical and promising application in the field of invasive interventional therapy. Schakenraad et al. [1] performed the first degradable polymer vascular stents using polylactic acid materials. Biodegradable polymer vascular stents are convenient for drug loading to prevent in-stent restenosis. The local drug concentration in the vascular wall and the duration of the drug release during drug release play a decisive role in the therapeutic effect. Clinical trials have shown that smooth muscle cell proliferation can be inhibited only when the drug concentration is within the threshold and for more than 30 days [2,3,4]. Many studies on the drug release process of drug loaded vascular stents are conducted through experiments [5,6,7,8,9,10,11,12]. However, the drug release and delivery of drug-loaded vascular stents is a complex process, which is affected by the drug-loading properties and degradation of drug-carrying polymers, as well as the three-dimensional structure of vascular stents [13].

The finite element method (FEM) is a convenient and effective method to study the drug release process of drug-loaded vascular stents. Naghipoor et al. [14] developed a two-dimensional degradable drug-eluting stent model to demonstrate the dependence of the drug profile on the porosity and degradation rate of the polymer and the dissolution rate of the drug. Wu Hao et al. [15], who established a three-dimensional model containing a vascular stent and arterial wall, studied and analyzed the concentration distribution and drug release of two drugs, paclitaxel and everolimus, within the vascular wall. Vijayaratnam et al. [16] used a two-dimensional computational fluid dynamics approach to simulate the drug transport and hemodynamics during a poor stent apposition. Vairo et al. [17] constructed a multi-domain two-dimensional model describing the drug transport in stent coatings, arterial tissue, and blood flow. Zhu et al. [18] proposed a mathematical model to describe the drug release process in PLGA-coated stents and the pharmacokinetics, and the simulation results showed that the internalization of the drug, interstitial fluid flow in the arterial wall, and stent pre-embedding had little effect on the amount of drug released. Guo et al. [19] provided a comprehensive multiscale computational framework to study the drug release of a dual-layer coating drug-eluting stent. Anna et al. [20] investigated the effect of the drug release on in-stent restenosis by computer methods based on the FEM. The present studies provide a numerical method for the calculation of the drug release from stents. However, the three-dimensional structure of the stent affects the intravascular hemodynamics, which can affect the drug release and delivery process in the vascular area. Thus, the influence of the three-dimensional structure of the stent needs to be considered in the drug release study of the vascular stent.

Polymers can effectively regulate the drug release rate during the release of drugs from drug-loaded stents [21,22]. PLGA-paclitaxel exhibits an extremely slow-release rate with a release time of several months [23,24,25], which is compatible with the desired drug release time. PLGA is widely used to fabricate biodegradable polymer stents due to its excellent biocompatibility, biodegradability, and controlled degradation rate [26], and is also used as a carrier for drug release. Apparently, the degradation properties of PLGA will change its porosity, which will affect the drug diffusion process in PLGA, and then affect the in-stent drug release characteristics. In 1998, Yamawaki et al. [27] developed the world’s first fully biodegradable drug-eluting vascular scaffold, Igaki-Tamai, with PLGA as the drug carrier. Mcgintyet et al. [28] compared the drug release process of permanent polymer coatings with that of biodegradable polymer coatings and concluded that biodegradable polymer coatings can better control the drug release for a therapeutic effect. Naghipoor et al. [29] used mathematical modeling and numerical simulation to simulate the drug slow-release process of PLGA polymeric vascular stents and proposed that the degradation factor results in a faster drug release and higher peak local drug concentration. Denny et al. [30] developed a numerical model to assess the effect of physical factors that promote or hinder drug release into the arterial wall on the drug slow-release process. Pontrelli et al. [31] proposed a mass diffusion model for drug-eluting stents to the arterial wall and demonstrated that the delayed drug release depends on the physical and chemical properties of the drug versus the microstructure of the polymer matrix. However, the degradation and solvation of degradable polymers are two completely different reaction processes. Degradation is a chemical reaction due to the breakage of polymer molecular chains, while solubilization is a physical phenomenon in which monomers and oligomers break away from the polymer. The slow-release rate of drugs in degradable polymer-loaded stents is due to the synergistic effect of the degradation and solubilization of the degradable polymer. Considering the synergy of both will make the prediction results more realistic.

In this study, a numerical study on the drug release process of biodegradable polymer drug-loaded vascular stents was proposed. The finite element method was used to simulate the drug release process of three-dimensional drug-loaded polymer vascular stents, with the effect of polymer degradation on the drug release process being considered. The coupled effects of polymer degradation and dissolution were considered in the degradation process of PLGA. The physiological processes such as the drug interaction with smooth muscle cells in the vascular wall, blood flow, plasma infiltration, and convection–diffusion processes of the drug in the vascular wall and lumen were also simulated. In addition, the effects of the stent geometry, initial drug concentration, and physical and chemical properties of the polymer on the drug release process were analyzed.

## 2. Method

**Figure 1:**
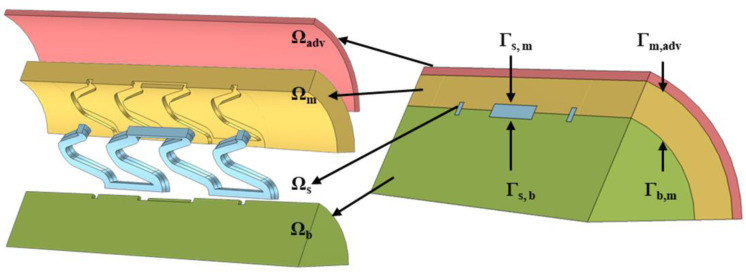
Three-dimensional geometry of the vascular stent and vessel wall for FEM. In which, $\Omega_{m}$, $\Omega_{adv}$, $\Omega_{b}$, and $\Omega_{c}$ represent the inner and middle layer regions of the vascular wall, the outer membrane layer region, the blood flow in the straight artery, and the biodegradable polymer-loaded stent, respectively. ${Г}_{s,m}$, ${Г}_{s,b}$, ${Г}_{b,m}$, and ${Г}_{m,adv}$ represent the boundary between drug-loaded stent and the middle layer, the boundary between drug-loaded stent and blood flow, boundary between blood flow and the middle layer, and the boundary between the outer membrane layer and the middle layer.

The finite element method was used to solve the drug release process of biodegradable polymer drug-loaded vascular stent in vivo (COMSOL Multiphysics 6.1, Burlington, MA, USA). The finite element model was shown in Figure 1. The vascular stent [32] was assumed to be implanted in the region of a straight artery. The geometric structure of the stent has a good symmetry property. Thus, three units of the stent were taken into account to simulate in-stent drug release process to reduce the amount of calculation, as shown in Figure 1. The dimensions of the vascular wall, stent, and blood basin are referenced in the literature [33]. The stent is embedded in the arterial wall to a depth of 50%. Since the inner and middle layers have similar properties for drug delivery [34], and the stent is often embedded in the inner layer, the vascular wall can be simplified into a two-layer structure in order to reduce the calculation. The inner and middle layers can be regarded as one layer named “middle layer”. The vascular wall is considered to be an anisotropic porous medium. As shown in Figure 1, $\Omega_{m}$, $\Omega_{adv}$, $\Omega_{b}$, and $\Omega_{c}$ represent the inner- and middle-layer regions of the vascular wall, the outer-membrane-layer region, the blood flow in the straight artery, and the biodegradable polymer-loaded stent, respectively. Γ represents the boundary of the adjacent region or interface. The subscripts m, adv, b, and s represent the middle layer, outer membrane layer, blood flow, and drug-loaded stent, respectively. In addition, the degradation process of biodegradable polymer vascular stents is considered. The degradation process of polymer vascular stents can lead to changes in polymer porosity ε, which will affect the release rate of drugs within the polymer vascular stent. For detailed control equations and boundary conditions, see Appendix A.

## 3. Numerical Results

### 3.1. Drug Release in Biodegradable Polymeric Drug-Loaded Stents

**Figure 2:**
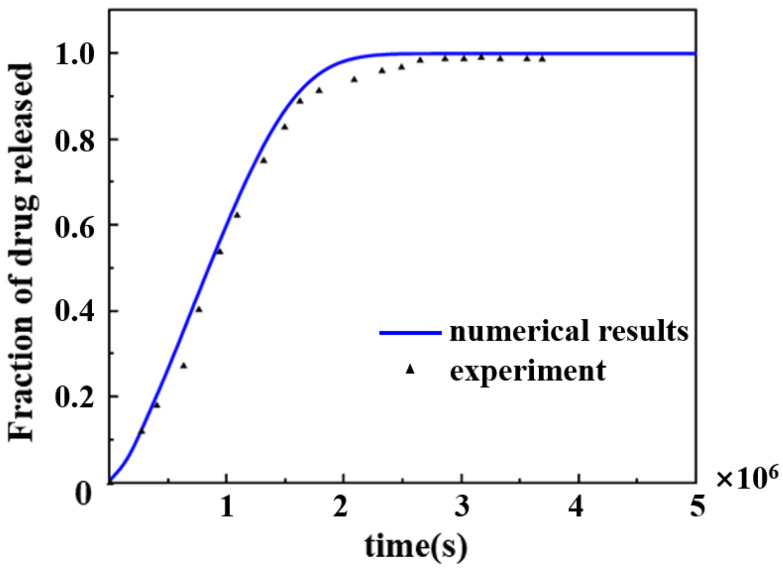
In vitro experimental data of sustained release of paclitaxel drug with PLGA as polymer matrix compared with the model data by finite element method this paper.

The results of the drug retardation predicted by the numerical model developed in this paper were compared with the results of in vitro experiments on the retarded release of paclitaxel using PLGA as the matrix by Lao et al. [35] (Figure 2), which verified the accuracy of the three-dimensional convective diffusion numerical simulation developed in this paper. The model predicted the percentage release of paclitaxel from the PLGA-based drug-carrying scaffold over 60 days, and the trend of the percentage drug release over 60 days was found to be in good agreement with the in vitro experimental results. The error between the model prediction and the experimental results was around 2 × 10^6^ s, which might be due to the difference in pH value of the buffer in the in vitro experiment and the experiment in this paper, and the different acid–base environment would lead to a slightly different release mechanism of paclitaxel.

**Figure 3:**
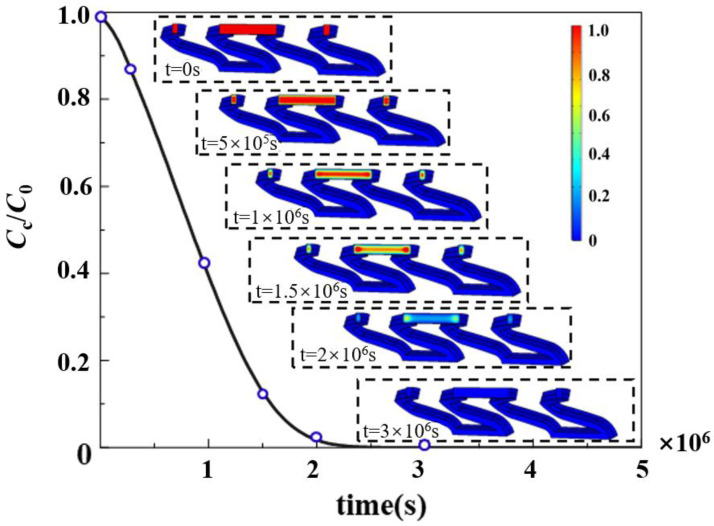
Drug concentration distribution and concentration variation in biodegradable polymeric drug-loaded stents. The distribution of drug concentration inside the stent at 5 typical time points is shown.

The distribution of the drug concentration and the variation in concentration values in the stent are shown in Figure 3. The drug release is divided into two phases: burst release and slow release. When the biodegradable polymer drug-loaded stent is implanted into the vascular area, the polymer boundary first contacts the blood and starts to swell, the blood encounters the solid drug particles through the pores so that they start to dissolve, and the liquid phase drug diffuses outside the polymer matrix. The degradation and dissolution of the polymer makes the pores larger and accelerates the drug release. As the drug release proceeds, the drug concentration in the outer layer of the stent decreases, the diffusion distance of drug release in the inner layer is relatively long, and the drug release rate gradually decreases.

### 3.2. Intravascular Pharmacokinetics

**Figure 4:**
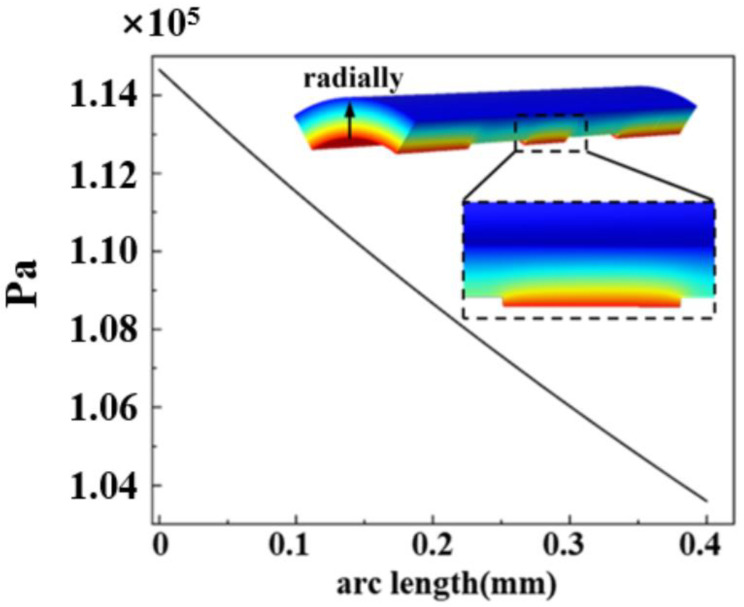
The pressure distribution within the vessel wall. The pressure within the blood vessel wall gradually decreases radially, which drives blood flow and drug diffusion and permeation within the vessel wall.

The pressure distribution within the vascular wall that facilitates drug delivery was shown in Figure 4. The maximum pressure within the vascular wall is located between the support units and decreases from inside to outside along the radial direction. The difference between the internal and external pressures drives the plasma penetration into the vascular wall and drives the drug release along the radial direction.

**Figure 5:**
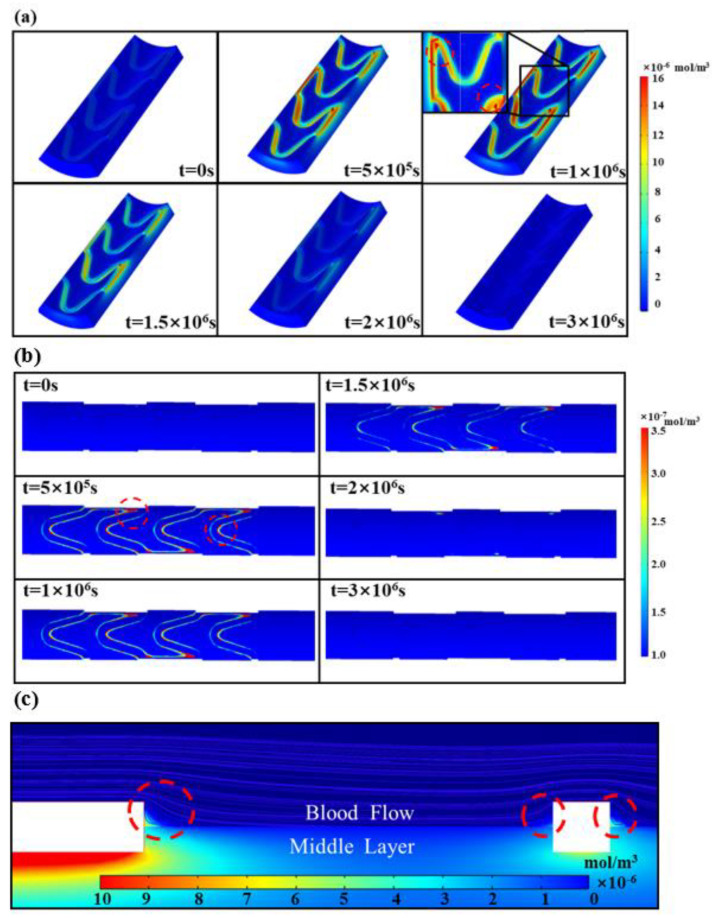
Drug release of biodegradable polymer drug-loaded stent in blood vessel: (**a**) changes in the distribution of drug concentration in the blood vessel wall at different moments; (**b**) changes in the distribution of drug concentration in the blood stream at different moments; and (**c**) drug deposition and vortex formation in the blood stream.

As shown in Figure 5a, the drug diffuses from the drug-loaded support matrix to the endothelial layer, and the drug concentration first gradually increases with time and then slowly becomes smaller. Moreover, it can be observed that the drug concentration in the middle layer of the vascular wall near the peak of the support unit and at the connection between the bridge tendon and the support unit is significantly higher than the other locations. The drug that diffuses into the blood stream flows away rapidly with the blood stream, but different levels of drug deposition were observed near the peak of the support unit and at the junction of the bridge tendon and the support unit (Figure 5b), and the drug concentration at the deposition site also showed a trend of increasing and then decreasing. The blood flow is blocked near the peak of the support unit and at the connection between the bridge bar and the support unit, and vortices are formed to generate negative pressure (Figure 5c). It makes the drug that diffuses into the blood stream form a drug pool here. Moreover, under the effect of a higher concentration difference and pressure difference, the drug concentration at the corresponding location in the vascular wall is greater than at other sites.

**Figure 6:**
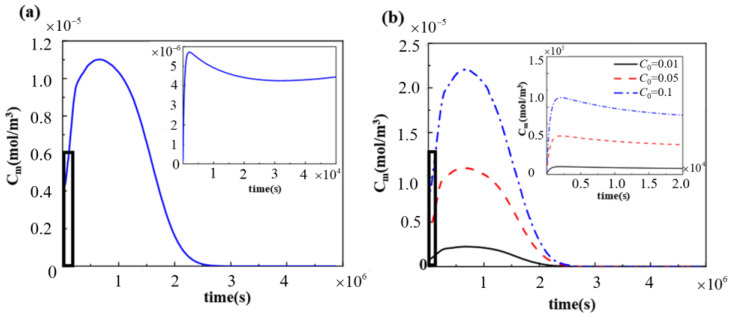
(**a**) Changes in the averaged drug concentration in the middle layer of the vessel wall; and (**b**) changes in the averaged drug concentrations in vascular wall at different initial drug concentrations, $C_{0}$ = 0.01; 0.05; and 0.10.

It is shown that only when the drug concentration reaches a certain threshold will it inhibit smooth muscle cell proliferation without toxic side effects [35,36,37], so the changes in drug concentration in the vascular wall should be concerned. The changes in the drug concentration in the middle layer of the vascular wall during drug release are shown in Figure 6a. A comparison of Figure 6a with the experimental results of Liu et al. [38] revealed that the changes in drug concentration in the middle layer of the vascular wall during drug release were basically the same. The average drug concentration is used to measure the drug concentration in each layer of the vascular wall, as shown in Equation (1).

(1) $${\bar{C}}_{i}=\frac{\int \Omega_{i}C_{i}(t)}{V_{\Omega_{i}}}$$

where $C_{i}$ is the drug concentration in layer i of the vascular wall, i=m, and Adv. During the burst release phase, the drug concentration in the vascular wall increases rapidly. During the slow-release phase, the drug release rate becomes smaller, the amount of drug that can be absorbed in the middle layer per unit time decreases, and the drug diffuses to the outer membrane layer and penetrates the bleeding tube wall, so the drug concentration in the middle layer decreases slightly. This result was also reflected in the study carried out by Cai et al. [38] on the use of a bilayer drug release system to carry pirfenidone for the manufacture of ureteral stent tubes for the prevention of ureteral strictures. However, the drug retardation rate then increased again under the synergistic effect of degradable polymer degradation and solubilization. Since the rate of drug release is gradually greater than the rate of drug exudation from the middle layer, the drug forms a buildup in the middle layer, so the concentration of the drug in the middle layer increases again. Finally, as the drug release ends, the intermediate-layer drug concentration gradually converges to 0, which was also mentioned by Zhuo et al. [39].

### 3.3. Effect of Drug Loading of Drug-Loaded Stent on Pharmacokinetics

The drug loading of the drug-loaded stent has a significant impact on the duration of drug release and the drug concentration in the vascular wall and blood flow. There are two ways to increase the drug loading. One is by increasing the initial drug concentration, and the other is by increasing the thickness of the drug-loaded stent matrix. Although both methods can increase drug loading, the effect on pharmacokinetics is different between the two.

**Figure 7:**
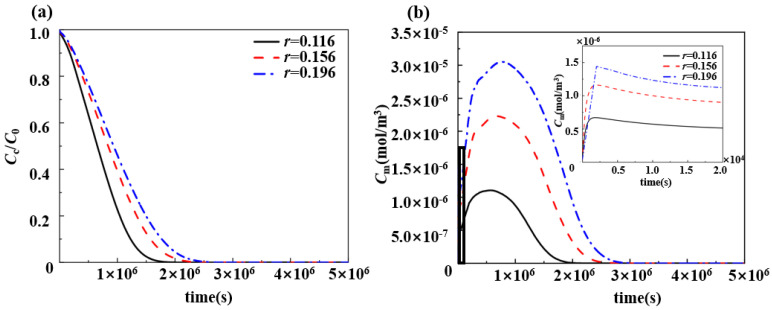
Effect of drug-carrying stent matrix thickness on drug slow-release process, where r is the drug-loaded stent matrix thickness, and $c_{c}$ and $c_{0}$ are the drug concentration and initial drug concentration in PLGA matrix, respectively: (**a**) changes in drug concentration in the drug-loaded stent matrix; and (**b**) changes in the averaged drug concentration in the middle layer of the vessel wall.

$c_{c}$ and $c_{0}$ are the drug concentration and initial drug concentration in the PLGA matrix, respectively. Therefore, $c_{c}/c_{0}$ can be used to describe the degree of drug release within the PLGA matrix. It can be seen from Figure 7 that, if the drug loading was varied by changing the thickness of the drug-loaded stent matrix, the drug release duration was changed. As the drug-loaded matrix thickness increased, the drug release time became longer, the drug release became slower, and the peak drug concentration in the vascular wall shifted backward (Figure 7a). Changing the initial drug concentration did have no effect on the drug release duration, but the peak drug concentration within the vascular wall increased with the increase in the initial concentration (Figure 6b). This characterization is also reflected in the findings of Cai et al. [38] and Zhuo et al. [39]. Furthermore, the increase in the thickness of the drug-loaded matrix also increased the peak drug concentration in the vascular wall with the same initial drug concentration (Figure 7b).

### 3.4. Effect of Porosity and Degradation Rate Coefficients of Polymers on the Pharmacokinetics

**Figure 8:**
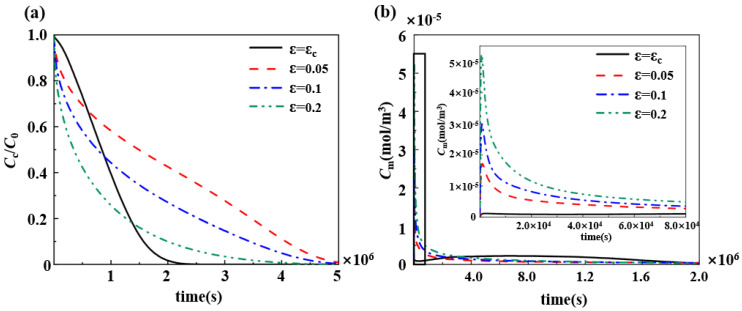
The difference between degradable and permanent polymers for pharmacokinetics: (**a**) changes in drug concentration in degradable polymer and permanent polymer drug-loaded stent matrices; and (**b**) changes in the averaged drug concentration in the middle layer of the vessel wall during drug retardation of biodegradable polymer and permanent polymer-loaded stents.

The porosity of the permanent polymer remained constant until complete drug release, while the porosity of the degradable polymer changed due to its self-degradation and dissolution, so the drug release rate trends were different for the two. The drug release process of the degradable polymer stents is divided into two phases, burst release and slow release, which is not available for the permanent polymer stents. As can be seen in Figure 8b, the permanent polymer stent releases the drug rapidly at the early stage of drug release and diffuses into the vascular wall, resulting in a rapid increase in drug concentration within the vascular wall. Although the duration of drug release of the permanent polymer stent is longer than that of the biodegradable polymer stent as seen in Figure 8a, the large amount of drug release at the early stage makes the permanent polymer stent unable to maintain high drug concentrations in the middle and late stages of drug release, and its effective treatment time is very short. In contrast, the degradable polymer stents have a burst release at the beginning of drug release, but, due to the change in their porosity, they can maintain a high drug concentration during the slow-release phase, which ensures the length of the effective treatment time.

**Figure 9:**
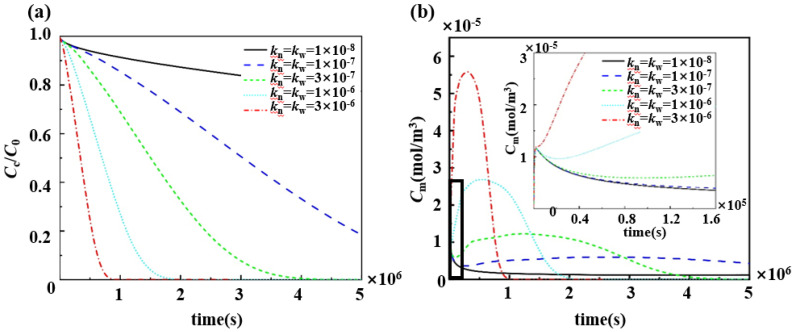
Pharmacokinetics of degradable polymeric drug-loaded stents with different degradation rate coefficients: (**a**) changes in drug concentration in the drug-loaded stent matrix; and (**b**) changes in the averaged drug concentration in the middle layer of the vessel wall.

The degradation rate of biodegradable polymers also affects the drug release trend, and the pharmacokinetics of five groups of degradable polymer stents with different degradation rate coefficients were compared (Figure 9). An increase in the degradation coefficient of the polymer resulted in an increase in the rate of drug release (Figure 9a). Furthermore, the change in polymer degradation coefficient also influenced the trend of the drug concentration within the vascular wall., According to previous studies, using biodegradable polymer stents to release drugs resulted in two peak drug concentrations in the vascular wall, which was also verified in three sets of simulations with degradation rate coefficients of $k_{n}$ = $k_{w}$ = 1 × 10^−7^, 3 × 10^−7^, and 1 × 10^−6^. However, we found that, when the degradation rate coefficient of the polymer was excessively large and small, both resulted in the disappearance of the peak in the slow-release phase (Figure 9b). When $k_{n}$ = $k_{w}$ = 1 × 10^−8^, after the burst release, the excessively small degradation rate limits the diffusion of the drug, so that the drug concentration in the vascular wall remains low and no concentration peak occurs. In contrast, when $k_{n}$ = $k_{w}$ = 3 × 10^−6^, the drug concentration in the vascular wall increased rapidly during the burst release phase. The excessive degradation rate causes the rapid degradation of the degradable polymer matrix and massive diffusion of the drug, resulting in the absence of the slow-release phase.

## 4. Discussion

### 4.1. Effect of Varying Drug Loading on the Slow-Release Process of Drugs

Many clinical trial data prove that the therapeutic success of drug-loaded stents depends not only on the drug itself but also on the drug concentration, and the drug release time plays a decisive role. When the concentration of the drug in the vascular wall is too high, it can lead to toxic side effects, mainly in the form of hematotoxicity, neurotoxicity, and hypersensitivity reactions. It has been shown that the therapeutic effect of using low doses of paclitaxel slow-release is substantial and that the effect of the release duration on the inhibition of smooth muscle cell proliferation is greater than the effect of the paclitaxel dose on it [40]. It follows that drug-loaded stents should be designed to ensure that the drug release time is as long as possible while the drug concentration in the vascular wall is within the threshold.

Although changing both the initial drug concentration and the thickness of the drug-loaded matrix can increase the drug load, the effects on the duration of the drug release, however, are very different for both. Changing the initial drug concentration with an unchanging diffusion path length only results in an increase in the peak drug concentration within the vascular wall and does not prolong the effective treatment duration. On the contrary, the effective treatment duration can be prolonged by increasing the thickness of the drug-loaded matrix. It is because the increase in the diffusion pathway reduces the drug diffusion rate, which shifts the peak drug concentration in the vascular wall during the slow-release phase and increases the drug release time. Therefore, when the drug load is certain, a smaller initial drug concentration and a larger stent thickness can provide an extended drug slow-release process at a low dose, resulting in a more pronounced therapeutic effect.

### 4.2. Adjustment of the Drug Slow-Release Process by Changing the Physical and Chemical Properties of the Polymer

Three generations of drug-loaded stents have been developed, the first two using permanent polymers as a matrix. As shown in Figure 7, the rapid drug release rate of permanent polymer stents makes the drug concentration within the vascular wall too large in the early stages and too small in the later stages, resulting in a short effective treatment time. More importantly, the excessive drug concentration within the vascular wall has the potential to cause toxic side effects. In contrast, the smaller initial porosity of the biodegradable polymeric drug-loaded stent inhibits the rate of drug release during the burst release, which results in a controlled drug concentration within the vascular wall. The variable porosity allows the drug concentration in the vascular wall to be maintained within the threshold value for about one month, and the drug release process is smooth and long-lasting. In addition, permanent polymers cannot be metabolized out of the body and have been shown to cause stent thrombosis, delayed healing, and inflammation in long-term clinical trials. In contrast, the matrix of third-generation drug-loaded stents was changed to a biodegradable polymer due to its good biocompatibility. The biodegradable polymers can eventually be hydrolyzed into small molecules and excreted from the body without causing toxic side effects. It can effectively reduce the chance of in-stent thrombosis and re-occlusion or restenosis after surgery.

The porosity of degradable polymers is influenced by the rate of degradation. The degradation rate is related to the molecular weight of the polymer, and the rate of change in molecular weight is determined by the degradation rate coefficient *k*_w_ [41]. A proper reduction of the degradation rate coefficient of the polymer can lead to a smooth and longer duration of the drug slow-release process. When the degradation rate coefficient is too small, the drug release rate is always smaller than the rate of drug dissipation by the vascular wall. Although the drug release time is prolonged, this may result in the drug concentration in the vascular wall always being less than the effective drug concentration and failing to inhibit smooth muscle cell proliferation. The excessive degradation rate factor makes the drug release process of drug-loaded stents similar to that of permanent polymeric stents. The rapid polymer degradation makes the drug release rate remain rapidly larger after the burst release, and an excessive amount of the drug accumulates in the vascular wall, risking toxic side effects and not ensuring sufficient effective treatment time.

The burst release of drug-loaded stents is unavoidable, and it has the risk of causing toxic side effects. In addition, the burst release reduces the duration of the subsequent slow-release phase, so the effective inhibition of the burst release is necessary. From the above results, it can be found that increasing the thickness of the drug-loaded matrix and decreasing the initial drug concentration can reduce the burst release time and the peak drug concentration in the vascular wall during the burst release phase. Taking the limit state of both, attaching a layer of an empty matrix outside the drug-loaded matrix seems to be an effective means to inhibit the burst release. Furthermore, the molecular weight of polymers can be changed by adjusting the ratio of the copolymers [41,42,43], thereby altering the degradation rate of polymers. Then, the use of degradable polymers with different degradation rates as drug delivery matrices also provides ideas for optimizing the therapeutic effect.

## 5. Conclusions

Based on numerical simulations, this paper investigates the pharmacokinetics of drug-loaded stents in blood vessels using a three-dimensional finite element method. In this study, the effects of stent matrix thickness, the physicochemical properties of the degradable polymer, and the initial concentration of the drug on the drug slow-release process of the degradable polymer-loaded stent with PLGA as the polymer matrix were systematically analyzed.

A three-dimensional computational model containing a biodegradable polymer drug-loaded stent, a blood basin, and a multilayer vascular wall structure was created. The model considers physiological processes such as the drug interaction with smooth muscle cells in the vascular wall, blood flow, plasma infiltration, and the convection–diffusion processes of drugs in the vascular wall and lumen, as well as the degradation and dissolution processes of the stent material. The distribution of drug concentrations within the vascular wall and the pattern of concentration changes were predicted by simulating the model.

Based on the numerical simulation of the drug slow-release process of the biodegradable polymeric drug-loaded stent, the effects of the stent matrix thickness, the physical and chemical properties of the degradable polymer, and the initial drug concentration on the pharmacokinetics were analyzed. The initial concentration of the drug influences the peak drug concentration in the intermediate layer of the vascular wall—the higher the initial concentration, the higher the peak—but the effect on the release time is negligible. The degradation and dissolution of degradable polymers cause changes in their porosity, resulting in a smoother and longer duration of drug release. The effect of the degradation rate coefficient of a polymer on the pharmacokinetics is complex, and too large or too small a degradation rate coefficient can lead to large changes in the pattern of drug concentration changes. When the degradation rate coefficient is moderate, the larger the degradation rate coefficient, the shorter the drug’s slow-release time, and the larger the peak drug concentration. When the drug concentration is certain, the thickness of the polymer matrix layer increases, the drug loading also increases, the slow-release rate of the drug becomes slower, and the peak drug concentration increases.

The study of the drug release properties and patterns is the basis for designing stent structures or drug carriers to improve drug release and, thus, the therapeutic efficacy of drug-loaded stents. The biochemical reactions and physiological environment dependent on drug transport in the vasculature are complex and not all of them have been considered in this paper. An assessment of the effects of other physiological phenomena on arterial drug uptake remains to be carried out in future work. However, it is hoped that the results of the study will be useful for the design of biodegradable polymer drug-loaded stents.

## Data Availability

The data underlying this article are available in the article.

## Appendix A. Control Equations and Boundary Conditions

When the drug-eluting stent encounters blood, the polymer swells and the drug encapsulated in the polymer begins to dissolve. Since the diffusion process of the drug after dissolution will be much slower than the dissolution itself, the autolytic phase of the drug in the polymer can be ignored. Here, it is assumed that the drug in the stent is completely dissolved into the liquid phase and the slow-release process of the drug in the coating can be controlled using the diffusion equation:

(A1) $$\frac{\partial C_{s}}{\partial t}+\nabla \cdot \left(-D_{s}\nabla C_{s}\right)=0$$

where $C_{s}$ is the drug concentration in the degradable polymeric stent and $D_{s}$ is the effective drug diffusion coefficient in the biodegradable polymer drug-eluting stents.

The drug release process in degradable polymer stents is coupled with the degradation and dissolution processes of degradable polymers. Zhu et al. [44] proposed a formula for the effective diffusion coefficient of drugs considering the degradation and dissolution of degradable polymers:

(A2) $$D_{s}=\frac{\left(1-\varepsilon_{s}\right)D_{d,pre}e^{\omega k_{n}t}+\beta_{s}\varepsilon_{s}D_{d,l}}{1-\varepsilon_{s}+\beta_{s}\varepsilon_{s}}$$

where $D_{d,pre}$ is the initial diffusion rate of the solid-phase drug before the degradation of the degradable polymer, $D_{d,l}$ is the diffusion rate of the liquid-phase drug in the matrix pores, and $\varepsilon_{s}$ is the porosity coefficient of the polymer change during the degradation process. $\beta_{s}$ is the drug partitioning between the liquid-filled pores and solid PLAG phase, and $k_{w}$ is the degradation rate coefficient associated with the change in the mass average molecular weight.

The porosity of the stent is described by the following equation:

(A3) $$\varepsilon_{s}=\varepsilon_{0}+\left(1-\varepsilon_{0}\right)\left(1+e^{-2k_{n}t}-2e^{-k_{n}t}\right)$$

where $\varepsilon_{0}$ is the initial porosity of the degradable polymer, assuming that the polymer matrix density is homogeneous and that the porosity of the undegraded matrix is 0. $k_{n}$ is the degradation rate coefficient related to the change in the number average molecular weight [45]. PLGA is a degradable polymeric material that remains structurally intact after the complete release of the drug, despite the reduction in quality due to degradation and solubilization [46]. Therefore, the shape of the polymer matrix is assumed to remain unchanged during the slow release of the drug.

Since smooth muscle cells have an elongated shape, drug diffusion in the vessel wall is hindered by radial diffusion; the axial diffusion rate of the drug is greater than the radial diffusion rate [47]. Hwang et al. [48] found that the relationship between the axial diffusion coefficient and radial diffusion coefficient of the vessel wall was $D_{z}$/$D_{r}=1$0. This ratio was used in this study.

The drug in the middle layer of the vessel wall reacts with receptors on the surface of smooth muscle cells through a metabolic process, thus acting as a therapeutic antiproliferative agent [49]. The drug delivery process in the middle layer of the vessel wall can be controlled by using the mean volume convective diffusion equation:

(A4) $$\frac{\partial C_{m}}{\partial t}+\nabla \cdot \left(-D_{m}\nabla C_{m}+C_{m}^{\prime }\gamma_{1}v_{p}\right)+\beta C_{m}=0$$

where $C_{m}$ is the concentration of the rug in the middle layer of the vessel wall, and $D_{m}$ is the effective diffusion coefficient of the drug in the middle layer of the vessel wall. The hindrance coefficient $\gamma_{1}\left(0<\gamma_{1}\leq 1\right)$ expresses the effect of friction between the drug molecules in the vessel wall and the pores of the vessel wall tissue. β is the first-order reaction rate coefficient, and $V_{p}$ is the average volume permeation rate of plasma in the tissue.

Using the same analytical approach, drug delivery in the outer membrane layer of the vessel wall and within the vessel lumen can be controlled using the convection–diffusion equation:

(A5) $$\begin{array}{l} \frac{\partial C_{adv}}{\partial t}+\nabla \cdot \left(-D_{adv}\nabla C_{adv}+C_{adv}^{\prime }\gamma_{2}v_{p}\right)=0\\ \frac{\partial C_{b}}{\partial t}+\nabla \cdot \left(-D_{b}\nabla C_{b}+C_{b}v_{b}\right)=0 \end{array}$$

where $D_{adv}$ and $D_{b}$ are the effective diffusion coefficients of the outer membrane layer and the vascular lumen, respectively, $\gamma_{2}$ is the obstruction coefficient of the outer membrane layer, and $V_{b}$ is the blood flow velocity in the vascular lumen.

Blood flow is considered as a homogeneous, isothermal, incompressible Newtonian fluid. The flow of blood within the vascular lumen can be controlled using the Navier–Stokes equations and the continuity equations as shown in Equation (A6).

(A6) $$\begin{array}{l} \rho_{b}\left(v_{b}\cdot \nabla \right)v_{b}-\nabla \cdot \left(\mu_{b}\nabla v_{b}\right)+\nabla p=0\\ \nabla \cdot v_{b}=0 \end{array}$$

where $\mu_{b}$ is the dynamic viscosity of blood flow, $V_{b}$ is the density of blood flow, and P is the pressure of blood flow in the lumen of the vessel.

Considering the effect of plasma convection, without considering the inhomogeneous structure of the arterial wall, which is considered a porous medium with various anisotropies and homogeneity, the plasma permeation process is controlled by Darcy’s law.

(A7) $$v_{w}+\frac{k_{w}}{\mu_{p}}\Delta P_{w}=0$$

where $\mu_{p}$ is the dynamic viscosity of the plasma, ${\begin{array}{c} v \end{array}}_{w}$ is the penetration velocity of the plasma, $k_{\begin{array}{c} w \end{array}}$ is the Darcy permeability coefficient of the arterial wall, △$p_{w}$ is the steady-state wall penetration pressure, $\Delta P_{w}=P_{b}-P_{e},\;P_{b}$ is the pressure in the lumen of the vessel, and p_e_ is the pressure at the outer boundary of the arterial wall.

Based on the previous assumptions and descriptions, the controlling equations for drug delivery in the drug-loaded stent, vessel wall, and vessel lumen are shown in Equation (A8), and the initial conditions for drug delivery in different regions of the vessel wall are shown in Equation (A9), and the boundary and interface conditions for drug delivery in different regions of the vessel wall are shown in Equation (A10).

(A8) $$\begin{array}{cc} \frac{\partial C_{s}}{\partial t}+\nabla \cdot \left(-D_{s}\nabla C_{s}\right)=0 & \;\mathrm{in}\;\Omega_{s}\times (0,T)\\ \frac{\partial C_{m}}{\partial t}+\nabla \cdot \left(-D_{m}\nabla C_{m}+C_{m}^{\prime }\gamma_{1}v_{p}\right)+\beta C_{m}=0 & \;\mathrm{in}\;\Omega_{m}\times (0,T)\\ \frac{\partial C_{adv}}{\partial t}+\nabla \cdot \left(-D_{adv}\nabla C_{adv}+C_{adv}^{\prime }\gamma_{2}v_{p}\right)=0 & \;\mathrm{in}\;\Omega_{adv}\times (0,T)\\ \frac{\partial C_{b}}{\partial t}+\nabla \cdot \left(-D_{b}\nabla C_{b}+C_{b}v_{b}\right)=0 & \;\mathrm{in}\;\Omega_{b}\times (0,T) \end{array}$$

(A9) $$\begin{array}{l} C_{s}=C_{0}\\ \;\mathrm{in}\;\Omega_{s},\Omega_{m},\Omega_{adv}\times (0)\\ C_{m}=C_{adv}=C_{b}=0 \end{array}$$

where $C_{0}$ is the initial drug concentration in the coating, assuming that the drug is uniformly distributed in the polymer matrix and completely dissolved into a liquid state before drug release (t = 0).

(A10) $$\begin{array}{cc} J_{s}\cdot n_{s}=-J_{b}\cdot n_{b},C_{s}^{\prime }=C_{b} & \;\mathrm{on}\;\Gamma_{s,b}\\ J_{s}\cdot n_{s}=-J_{m}\cdot n_{m},C_{s}^{\prime }=C_{m}^{\prime } & \;\mathrm{on}\;\Gamma_{s,m}\\ J_{m}\cdot n_{m}=-J_{b}\cdot n_{b},C_{m}^{\prime }=C_{b} & \;\mathrm{on}\;\Gamma_{m,b}\\ J_{m}\cdot n_{m}=-J_{adv}\cdot n_{adv},C_{m}^{\prime }=C_{adv}^{\prime } & \;\mathrm{on}\;\Gamma_{m,adv} \end{array}$$

where it is assumed that the drug concentration and flux are continuous at the interface between the layers of the vessel wall, and *i* = m, adv, b, and s.

(A11) $$J_{s}=-D_{s}\nabla C_{s},J_{m}=-D_{m}\nabla C_{m}+\frac{C_{m}\gamma_{1}}{k_{m}\varepsilon_{m}}\cdot v_{p},J_{adv}=-D_{adv}\nabla C_{adv}+\frac{C_{adv}\gamma_{2}}{k_{adv}\varepsilon_{adv}}\cdot v_{p},C_{i}^{\prime }=\frac{C_{i}}{k_{i}\varepsilon_{i}}$$

The physiological pressure difference between the inside and outside of the vessel wall is approximately 100 mm Hg. The controlling equation for blood flow in the lumen and plasma permeation in the vessel wall can be described as follows:

(A12) $$\begin{array}{l} \rho_{b}\left(v_{b}\cdot \nabla \right)v_{b}-\nabla \cdot \left(\mu_{b}\nabla v_{b}\right)+\nabla p=0,\;\nabla \cdot v_{b}=0\\ v_{w}+\frac{k_{w}}{\mu_{p}}\Delta p_{w}=0,\nabla \cdot v_{p}=0\\ P_{b}=860\mathrm{mmHg}\;\mathrm{on}\;\Gamma_{m,b}\\ P_{e}=760\mathrm{mmHg}\;\mathrm{on}\;\Gamma_{0} \end{array}$$

Describe the inlet flow rate in the conduit lumen with a Poiseuille flow curve.

(A13) $$\bar{\nu }=\nu_{\max}\left(1-{\left(\frac{r}{R}\right)}^{2}\right)$$

where $V_{max}$ is the maximum blood flow velocity, R is the lumen radius, and r is the radial position of the blood basin.

The parameters applied in the numerical simulation are detailed in Table A1 [49,50,51,52,53,54].

**Table A1:** Values for the parameters applied in numerical analyses.

**Model Domain**	**Parameters**	**Reference Value**
The blood basin	Vascular wall radius, R (mm) Blood viscosity, $\mu_{b}$ (g/cm·s) Density, $\rho_{b}\;(g/{\mathrm{cm}}^{2})$ Maximum flow rate at the entrance, $v_{max}\;(m/s)$ Paclitaxel diffusion coefficient, $D_{b}$ (m^2^/s)	1.5 3.5 × 10^−2^ 1.057 0.14 10^−10^
Blood vessel wall	Thickness of the middle layer, rm (mm) Thickness of the outer layer, radv (mm) Plasma viscosity, $\mu_{p}$ (g/cm·s)	0.4 0.1 0.72 × 10^−2^
Drug-loaded stents	Diffusion coefficient of paclitaxel in biodegradable polymer drug-loaded stent, $D_{d,pre}\;(m^{2}/s)$ The paclitaxel liquid phase diffusion coefficient, $D_{d,l}\;(m^{2}/s)$ Drug partition coefficient, $\beta_{s}$ Correlation of diffusion coefficient to polymer molecular weight, α	10^−17^ 5 × 10^−11^ 10^−4^ 1.714
Middle layer	$\mathrm{Porosity},\;\varepsilon_{m}$ $\mathrm{Darcy}\;\mathrm{permeability},\;\mathrm{km}\;(m^{2})$ $\mathrm{Radial}\;\mathrm{diffusion}\;\mathrm{coefficient}\;\mathrm{of}\;\mathrm{paclitaxel},\;\mathrm{Dm},\;r\;(m^{2}/s)$ $\mathrm{Axial}\;\mathrm{diffusion}\;\mathrm{coefficient}\;\mathrm{of}\;\mathrm{paclitaxel},\;\mathrm{Dm},\;z\;(m^{2}/s)$ Drug partition coefficient, km Drug hindrance coefficient, γ1 Drug consumption coefficient, β (s^−1^)	0.61 2 × 10^−18^ 2.6 × 10^−12^ 2.6 × 10^−11^ 20 1 10^−4^
Outer film layer	$\mathrm{Porosity},\;\varepsilon_{Adv}$ $\mathrm{Darcy}\;\mathrm{permeability},\;k\;\mathrm{Adv}\;(m^{2})$ $\mathrm{Radial}\;\mathrm{diffusion}\;\mathrm{coefficient}\;\mathrm{of}\;\mathrm{paclitaxel},\;\mathrm{Dadv},\;r\;(m^{2}/s)$ $\mathrm{Axial}\;\mathrm{diffusion}\;\mathrm{coefficient}\;\mathrm{of}\;\mathrm{paclitaxel},\;\mathrm{Dadv},\;z\;(m^{2}/s)$ Drug partition coefficient, kadv Drug hindrance coefficient, γ2 Outer boundary permeability, P0 (m/s)	0.85 2 × 10^−18^ 4 × 10^−12^ 4 × 10^−11^ 20 1 10^−6^
